# Direct Immunofluorescence of Skin and Oral Mucosa: Guidelines for Selecting the Optimum Biopsy Site

**DOI:** 10.3390/dermatopathology11010006

**Published:** 2024-01-19

**Authors:** Muhammad N. Mahmood

**Affiliations:** Department of Laboratory Medicine and Pathology, University of Alberta Hospital, Edmonton, AB T6G 2B7, Canada; mmahmood@ualberta.ca; Tel.: +1-780-407-2145

**Keywords:** direct immunofluorescence, biopsy site, vesiculobullous disorder, vasculitis, connective tissue disease, oral mucosa, transport media

## Abstract

Direct immunofluorescence is a vital diagnostic test for assessing vesiculobullous disorders, vasculitides, and connective tissue diseases. It is a robust and valuable technique that offers essential diagnostic information for many critical dermatoses. Dermatopathologists depend heavily on the data obtained from direct immunofluorescence evaluation to confirm final diagnoses. Selecting the most appropriate biopsy site is necessary for maximizing diagnostic accuracy, and the best site may vary depending on the clinical differential diagnosis. Inaccurate biopsy site selection can significantly impact the accuracy of the results. To optimize the use of direct immunofluorescence studies, this review provides helpful guidelines and some practical tips for selecting the best biopsy site.

## 1. Introduction

Laboratory immunodermatology studies provide an excellent set of tools that can help diagnose various skin conditions. Direct immunofluorescence (DIF) is the primary diagnostic ancillary test used to evaluate vesiculobullous disorders (VBD), vasculitides, and connective tissue diseases (CTD). This technique targets abnormal proteins in the patient’s tissue using antibody–fluorophore conjugates. Compared to indirect immunofluorescence and enzyme-linked immunosorbent assay, DIF is more sensitive in detecting bullous pemphigoid (BP) [1]. It is a powerful and practical technique that provides a high level of diagnostic information for many critical dermatoses often seen among in-patients in the hospital setting. However, if not performed accurately, it can lead to erroneous diagnostic results. Pre-analytical factors such as suboptimal biopsy site selection, incorrect transport medium, delayed pathological processing, and improper slide storage can influence interpretation and results [2].

Dermatopathologists heavily rely on information gathered from DIF studies to establish final diagnoses. However, it can be challenging when they cannot confidently interpret a case due to pre-analytic or technical shortcomings. Choosing the optimal biopsy site for DIF studies is crucial to maximize diagnostic accuracy [2,3]. The preferred biopsy site varies depending on the conditions included in the clinical differential diagnosis. Poor site selection may lead to false negative or false positive DIF results. During their early years of training and practice, dermatology residents and other clinicians who do not regularly collect biopsy samples for DIF may have a practice gap in choosing the appropriate biopsy site. This contribution aims to provide helpful guidelines and practical tips that can aid physicians in determining the best biopsy site and optimizing the utilization of DIF. This article separately discusses the optimal skin or oral mucosal site for DIF in VBD, vasculitides, and CTD.

## 2. Vesiculobullous Disorder

Direct immunofluorescence is a crucial diagnostic tool for autoimmune VBD. A 4 mm punch biopsy is the recommended method for DIF. If a shave biopsy must be done, it should be deep enough to include the reticular dermis. In the case of BP, DIF biopsy is traditionally taken from perilesional skin, and the center of active blisters and distant uninvolved or never-involved sites are avoided [2,3]. Anstey et al. concluded in their study that a single perilesional biopsy is usually sufficient to provide results in both pre-treatment and post-treatment cases of BP [4]. More recently, Sladden et al. demonstrated a higher probability of positive DIF results in BP from lesional non-bullous (urticarial or pre-bullous) skin than perilesional or normal skin [5]. Other techniques have been suggested for subepidermal VBD, where the biopsy is taken from the edge of an active blister to contain part perilesional skin and part bullous lesion [6]. This technique can provide a bonus salt-split-like analysis in the bullous zone; however, if not correctly executed, the epidermis can completely separate from the dermis and compromise the evaluation.

For BP and other autoimmune subepidermal VBDs (i.e., pemphigoid gestationis, lichen planus pemphigoides, epidermolysis bullosa (EB) acquisita, bullous lupus erythematosus (LE), and linear IgA bullous dermatosis), all the approaches mentioned above can provide the desired results. The recommended site of DIF biopsy is perilesional erythematous or uninvolved skin, about 3 mm away from the edge of a blister but no more than 10 mm away, or non-bullous lesional skin, or the edge of an active blister (2/3 perilesional and 1/3 lesional). If in doubt, two biopsies can be taken for DIF, one from the blister’s edge and the other from perilesional erythematous or uninvolved skin. Also, the trunk and flexural skin of the forearms are generally preferred.

In the pemphigus group, the convention is to perform a DIF biopsy on perilesional erythematous or uninvolved skin, about 3 mm away from the edge of a blister but no more than 10 mm away. In addition, positive DIF results have been seen with high sensitivity utilizing the outer root sheaths of hair in patients with pemphigus [7]. For this economical, non-invasive technique, anagen hairs from the scalp are forcibly plucked using simple forceps, and immunofluorescence studies are performed on the outer root sheath.

Direct immunofluorescence results are crucial in diagnosing dermatitis herpetiformis (DH). Zone et al. found more intense IgA staining in perilesional normal-appearing, uninvolved skin compared to erythematous skin and distant, never-involved skin [8]. Their study defined perilesional skin as normal-appearing skin located 1 mm to 10 mm from a papulovesicle or area of erythema. The recommended site of DIF biopsy in DH is perilesional normal-appearing, uninvolved skin about 3 mm from the edge of the lesion. If IgA DIF results are negative, but there is a high clinical suspicion of DH, a repeat biopsy for DIF should be considered [9].

When evaluating porphyria and drug-induced pseudoporphyria, it is best to utilize lesional skin for DIF. Skin from the hands or face is preferred, and the biopsy is taken from the edge of an active blister to contain 1/3 perilesional skin and 2/3 lesional skin.

## 3. Oral Mucosal Biopsy for Vesiculobullous Disorder

The definitive diagnosis of oral mucosal autoimmune VBD requires DIF analysis. However, due to the fragility of the mucosa and the challenging access to certain parts of the mouth, the biopsy procedure can be complicated. As a result, it is advisable to reserve DIF biopsy only for patients with a strong clinical suspicion of autoimmune VBD [10]. Elliptical/wedge incisional scalpel and punch biopsies are utilized, with punch biopsy providing better diagnostic yield and sensitivity [11]. It is crucial to handle the biopsy gently, as mucosal tissue is easily susceptible to crush artifacts. It is vital to obtain a sample that retains its structural integrity with the epithelium attached to the subepithelial connective tissue.

When performing a biopsy of oral VBD for DIF, the traditional practice is to take a perilesional mucosa sample, about 3 mm away from the area of a blister or ulceration but no more than 10 mm away [12,13]. However, normal-looking non-lesional mucosa more than 10 mm away from the lesions can also provide accurate results. A study of a large group of patients with oral pemphigus vulgaris and multi-site mucous membrane pemphigoid (MMP) showed that a 4 mm punch biopsy of normal buccal mucosa delivers DIF results with sensitivity equivalent to perilesional biopsy [14]. This suggests that the immunoreactants may be present in the affected and unaffected oral mucosa. Other analyses confirm that normal-looking mucosa displays similar rates of DIF positivity to perilesional mucosa for MMP; however, perilesional mucosa shows slightly better results for oral pemphigus vulgaris [15]. Normal buccal mucosal punch biopsy offers an uncomplicated and sensitive method that various physicians can dependably employ, particularly in cases where the perilesional sites are technically challenging to access [16]. Due to chronic inflammation and fragility, the gingiva is considered an inferior biopsy site and should be avoided. For localized gingival MMP, neighboring normal reflected alveolar mucosa can be biopsied for DIF.

Performing multiple and repeated biopsies can increase the sensitivity of DIF in diagnosing MMP [17]. To achieve an accurate diagnosis, it is recommended to perform two biopsies for DIF, one from the perilesional area and the other from normal-appearing buccal mucosa. However, this approach can sometimes be redundant and impractical and should only be used selectively. Diagnosing ocular MMP can be challenging since up to 50% of cases do not meet the immunopathological criteria necessary for the diagnosis. This often leads to delayed diagnosis and poor outcomes for patients. To improve the sensitivity of DIF in complex cases, it is suggested to perform DIF biopsies of both bulbar conjunctiva from non-inflamed areas and normal buccal mucosa [18]. As the DIF of buccal mucosa can be positive even when the conjunctiva is negative, this approach can help establish an accurate and timely diagnosis.

## 4. Hereditary Epidermolysis Bullosa

Inherited EB is diagnosed and subtyped with the help of immunofluorescence antigen mapping and transmission electron microscopy. However, testing existing blisters (over 12 h old) is not recommended due to proteolytic antigenic degradation and artifacts caused by reepithelization. Instead, it is suggested to induce a fresh blister for testing purposes. Normal intact skin is selected, preferably neighboring where the patient usually gets blisters. The upper inner arm skin is preferred for this purpose, while the glabrous skin of palms and soles is avoided.

To induce a fresh blister, the skin is rubbed using a pencil eraser (or gloved finger, or cotton swab) with firm downward pressure followed by rotating it 180 degrees in each direction 3 to 5 times [19]. The skin is rubbed at least twenty times until it turns red. After the skin turns red, pause for about 5 min to allow for the development of a microscopic blister. Two punch biopsies are performed from the red area, one for immunofluorescence antigen mapping (submit in Michel’s or Zeus medium) and the second for electron microscopy (submit in 2.5% glutaraldehyde solution). If a blister develops at the site of rubbing, execute the biopsies so that each sample contains part of the blister’s edge and part of the perilesional skin (i.e., 2/3 perilesional and 1/3 lesional). In severe cases with extreme skin fragility, rubbing may not be required as the cleavage plane may form simply by the twisting motion of the punch biopsy procedure. It is recommended to avoid topical anesthetics as they may cause artefactual blistering, and injectable anesthetics are preferred.

It is important to note that these tests for hereditary EB are not routine at all laboratories and are frequently sent to specialized centers. As standards of specimen acceptance may vary, before scheduling a biopsy, the clinician should collaborate with the laboratory regarding additional collection and transport-related instructions [20].

## 5. Cutaneous Vasculitis

When it comes to diagnosing cutaneous vasculitis, the timing and location of the biopsy are critical factors for accurate interpretation. In immune complex-mediated vasculitis, diagnostic immunofluorescence highlighting immunoreactant deposition is inversely related to the duration of the lesions [21,22]. Immunoglobulin deposits tend to fade in older purpuric lesions and the highest percentage of biopsies showing the presence of immunoglobulins are observed within the initial 48 h after the lesion first appears. Between 48 and 72 h, the percentage of biopsies that show immunoglobulin positivity decreases to 70%, and after 72 h, no immunoglobulins are detected. Complement deposition lasts longer than immunoglobulins, with more than 50% of cases still showing positivity after 72 h. A study by Nandeesh and Tirumalae demonstrated that biopsy timing can affect the positivity of DIF in vasculitis [23]. They found that 85% of biopsies performed within the first 7 days were positive, 14% were positive between 1 and 2 weeks, and only 1% were positive between 2 weeks and 1 month.

In a study conducted by Giangiacomo and Tsai, 14 pediatric patients suffering from Henoch–Schönlein purpura were analyzed for DIF of purpuric lesional and normal-appearing uninvolved skin [24]. The study found that 93% of the biopsies from the lesional skin displayed vascular IgA positivity compared to only 43% of the biopsies from uninvolved skin, with half of the positive cases from uninvolved skin showing only weak staining. However, a study by Van Hale et al. displayed comparable vascular IgA positivity between lesional and uninvolved skin [25]. 

In vasculitis, the biopsy for DIF should be obtained from the edge or active border of a fresh pink blanchable macule, as immunoglobulins degrade with time in the center of older lesions [26]. The lesion selected should be newly evolved, ideally between 8 and 24 h, and no older than 48 h. DIF should not be performed on old necrotic or ulcerated lesions and hemorrhagic blisters. It is suggested to avoid sampling from distal lower legs if the patient has long-standing venous stasis-induced changes [27]. Normal-appearing uninvolved skin adjacent to lesions can also provide desired DIF results; however, it has a low sensitivity and high false negative rate.

## 6. Connective Tissue Disease

Direct immunofluorescence is used to investigate CTD, particularly the various expressions of LE. CTD is a diverse group of disorders with distinct clinical presentations, histopathological features, and immunological profiles. The immunological changes lead to autoantibodies, immune complexes, and immune deposits in the skin. Serological tests can identify the existence of circulating autoantibodies, while DIF can determine cutaneous immune deposits. Although DIF has been used to diagnose CTD, serological testing is considered more reliable. In fact, with the availability of improved serum serological tests, DIF may be unwarranted and only required in selected cases.

The sensitivity of DIF results is affected by various factors in LE, including clinical morphology, the duration of lesions, biopsy location, and the patient’s treatment status. As the lesion’s age increases, the frequency of positive DIF also rises. DIF positivity is higher in untreated lesions than in treated ones [28]. Treatment up to three weeks before biopsy reduces the frequency of DIF positivity. The positivity rate is also influenced by the location of the biopsy site on the body, with sun-exposed skin showing a higher frequency of DIF positivity than sun-protected skin. Sun-exposed healthy skin of up to 20% of normal adults can show non-specific false-positive staining at the dermoepidermal junction [29].

For evaluating CTD, lesional and uninvolved non-lesional sun-protected skin can be utilized for DIF analysis. In cases where discoid LE, subacute cutaneous LE, or dermatomyositis are suspected, a biopsy is taken from the erythematous active border of an established lesion. To ensure accurate results, the lesion should be older and have a duration of at least three months. Clinically, a ‘red and angry’ lesion is targeted, while ‘burnt-out’ lesions are avoided. An untreated lesion from an area not chronically exposed to sunlight is preferred if possible. 

In cases where systemic LE or acute cutaneous LE is suspected, two biopsies for DIF are obtained. One biopsy is taken from the erythematous active border of an established lesion, while the second biopsy for the lupus band test is obtained from non-lesional sun-protected skin like the buttocks, abdomen, and inner thigh [30]. It is worth noting that the DIF of perilesional skin does not provide any additional value in CTD diagnosis.

## 7. Lower Extremity Biopsy for Direct Immunofluorescence

When conducting a biopsy to evaluate BP, avoiding the lower extremity as the biopsy site is currently recommended. It is believed that biopsies from the lower extremities have a higher false negative rate of DIF in cases of BP. However, the evidence supporting this perception is lacking. The initial reference to avoid lower extremities is based on a study conducted around forty years ago. In this study, Weigand retrospectively analyzed 46 BP patients and found a DIF false negative rate of 33% on the lower extremities [31]. However, in a later study, Weigand and Clements re-evaluated this opinion [32]. They concluded that weak or false negative DIF results correlated with the extent of the disease (localized versus generalized) rather than the specific anatomic site of the lower extremity. In a 2018 study, Fudge et al. found no false negative DIF results on lower extremities in their series of BP patients [33]. In a retrospective study of 79 patients conducted in 2021, Perry et al. found no statistically significant difference in the DIF false negative rate based on the anatomical site of the biopsy [34]. Although the notion of avoiding lower extremities for the DIF evaluation of BP is often quoted, the supporting evidence is inconsistent and contradictory.

Regarding the DIF evaluation of vasculitis, a non-specific vascular deposition of immune complexes can occur on the distal lower extremities in venous stasis and certain panniculitides (e.g., erythema nodosum) [27]. As vasculitis preferentially involves the lower extremities, a biopsy of the lower extremities is often unavoidable and adds to the lack of specificity of DIF in vasculitis. To avoid false positives, the recommendation is to sample from the most proximal part of the lower limb and avoid distal lower extremities around the ankles. 

## 8. Limitations in Guidelines of Optimum Site

Most data vis à vis the ideal biopsy site for DIF are founded on retrospective analyses. The definitions of perilesional and lesional are often not standardized in the studies, and the data collected are predominantly built on the description provided by the clinicians. This introduces significant variability and imprecision regarding the actual site sampled. In routine dermatopathology practice, it is not uncommon to see a DIF biopsy designated as perilesional representing lesional skin and vice versa. The distance from the lesion or blister is sometimes used arbitrarily and is quoted differently by available resources. In vasculitis, the time of the lesion’s inception (e.g., less than 24 h or 48 h) is often based on a general estimation which may not be precise and often not available in the clinical information provided to the pathologist.

When it comes to biopsy sites for DIF in VBD, most of the information available is based on studies conducted on patients with BP. The recommendations established for BP patients are applied to other VBDs with entirely different pathophysiologies. In routine practice, the clinical presentation can be atypical, and there may be several diverse disorders in the differential diagnosis list. Therefore, choosing the best biopsy site requires a combination of the physician’s overall clinical judgment and the guidelines to select the most appropriate site. If the clinicians performing the biopsy have a good understanding of the fundamental concepts of selecting the site, they are more likely to select the site that will provide the best diagnostic information.

## 9. Transport Media and Handling

The proper transportation of the DIF biopsy is crucial for obtaining accurate results. Every laboratory has a specific protocol for transporting and storing biopsy specimens. The tests are optimized according to the set protocol, and constant changes in the transport media and specimen delivery process can lead to errors. Therefore, before performing the DIF biopsy, the clinician should ensure that the laboratory’s protocol and standards are followed and that a proper transport medium is available. A proper transport medium is critical as it prevents autolysis and maintains the sample’s antigenicity.

There are various ways to transport biopsy samples for DIF analysis. Some common modes include special transport media (e.g., Michel’s or Zeus medium), normal saline solution (0.9% NaCl), or saline-soaked gauze [35,36]. The samples can also be transported snap-frozen in liquid nitrogen. If normal saline is used, it is reliable only if the sample is received and processed within 24 h after the biopsy. If the tissue is transported frozen, it should not be thawed. Sometimes, samples are not processed over weekends or holidays, and the laboratory accessions Friday evening specimens on Monday. Further, the specimen collection site may transport the sample to a separate centralized immunohistochemistry laboratory for processing. These delays and logistic complications in specimen collection and processing can affect the diagnostic accuracy of frozen samples and those received in normal saline. Michel’s pH-neutral buffered ammonium sulfate solution is the preferred transport medium for DIF biopsies. Special transport media like Michel’s have an advantage over other methods as they preserve and safely store tissue over many days. Ensuring the biopsy is fully submerged in the medium is crucial; the biopsy should not adhere to the container’s cap or sides. Check the expiration date of the reagents to ensure that you do not use any that have already expired.

It is important to note that sending samples in 10% buffered formalin solution or dried in an empty container is inadequate [37]. In cases of pemphigus, formalin exposure of even 2 min can lead to negative results. DH and BP, on the other hand, retain staining after 10 min of exposure to formalin; however, after this point, the intensity of positivity starts to decrease. Moreover, prolonged formalin exposure can create a non-immunologic nuclear fluorochrome pattern, interfering with other disease-specific staining or being misinterpreted as an in vivo antinuclear antibody reaction of LE.

To ensure that the specimen’s integrity is not compromised, it is essential to handle it correctly. When obtaining samples for cases requiring DIF, taking at least two punch biopsies is best. Paired samples optimize the diagnostic yield; one biopsy should be used for routine light microscopy, and the other should be reserved for DIF analysis. Bisecting a single punch biopsy has been suggested, but this technique has limitations in routine practice [6,38]. A larger punch (6 or 8 mm) is preferred if a single biopsy needs to be pre-bisected. The poor execution of the bisection can quickly induce a crush artifact and disrupt the blister. Mechanically manipulating the blister can cause the epidermis to separate entirely from the dermis, compromising both routine light microscopy and DIF interpretations. While carefully pre-bisecting a single biopsy may work in some subepidermal VBD, it is not suitable for intraepidermal VBD (e.g., pemphigus), hereditary EB, and Stevens–Johnson syndrome. In the latter conditions, as the blisters can be superficial, fragile, or have necrotic epidermis, manipulating the biopsy can easily disrupt its integrity. 

## 10. Conclusions

Regarding diagnostic accuracy, selecting the appropriate biopsy site is of the utmost importance. To ensure that the biopsy results are reliable and accurate, it is recommended that the biopsy site for DIF is determined carefully. A synopsis of these recommendations is provided in Table 1. It is noteworthy that poor site selection and improper specimen handling lead to errors in results and suboptimal evaluation, as highlighted in Table 2. Therefore, it is crucial to consider these factors while choosing the biopsy site and handling the specimen to avoid any discrepancies in the final diagnosis. Selecting the optimal biopsy site requires integrating the physician’s comprehensive clinical assessment and adherence to the criteria for determining the most suitable location. If the healthcare providers conducting the biopsy have a sound understanding of the critical principles of selecting a site, they are more likely to identify the location that will provide the most accurate diagnostic information.

## Figures and Tables

**Table 1:** Recommendations for direct immunofluorescence and immunofluorescence mapping biopsies.

**Vesiculobullous Disorder: Skin**
**- Bullous pemphigoid and other autoimmune subepidermal vesiculobullous disorders** • Perilesional erythematous or uninvolved skin, about 3 mm away from the edge of a blister but no more than 10 mm away; • Non-bullous lesional skin; • Edge of an active blister (2/3 perilesional and 1/3 lesional); • Trunk or flexural forearms preferred. **- Pemphigus group** • Perilesional erythematous or uninvolved skin, about 3 mm away from the edge of a blister but no more than 10 mm away; • Outer root sheath of anagen hair. **- Dermatitis herpetiformis** • Perilesional normal-appearing uninvolved skin, about 3 mm from the edge of the lesion; • Consider repeat biopsy for negative cases with high clinical suspicion. **- Porphyria and drug-induced pseudoporphyria** • Edge of an active blister (1/3 perilesional skin and 2/3 lesional); • Hands or face preferred. **- Hereditary epidermolysis bullosa** • To induce a fresh blister, normal-appearing uninvolved skin is rubbed using a pencil eraser; • Avoid existing blisters and skin from palms and soles; • Upper inner arm preferred.
**Vesiculobullous Disorder: Oral Mucosa**
**- Multi-site mucous membrane pemphigoid and oral pemphigus** • Perilesional mucosa, about 3 mm away from the edge of a blister but no more than 10 mm away; • Normal-appearing buccal mucosa; • Avoid gingiva. **- Localized gingival mucous membrane pemphigoid** • Perilesional mucosa, normal-appearing neighboring reflected alveolar mucosa. **- Ocular mucous membrane pemphigoid** • Two biopsies: non-inflamed bulbar conjunctiva, and normal-appearing buccal mucosa.
**Cutaneous vasculitis**
• Edge or active border of newly evolved lesion, between 8 and 24 h but no older than 48 h; • Avoid old necrotic or ulcerated lesions, hemorrhagic blisters, and distal lower legs around ankles.
**Connective tissue disease**
**- Discoid or subacute cutaneous lupus erythematosus and dermatomyositis** • Erythematous active border of established older lesion, duration of at least 3 months; • Avoid ‘burnt-out’ and treated lesion. **- Systemic or acute cutaneous lupus erythematosus** • Two biopsies: erythematous active border of established older lesion, and non-lesional sun-protected skin (e.g., buttocks, abdomen, or inner thigh).
**Common factors**
• Use appropriate, unexpired transport medium; • Check laboratory collection and transport-related instructions; • Multiple or repeat biopsies in complex cases; • Specify exact site of biopsy and patient’s treatment status on pathology requisition.

**Table 2:** Causes of errors or suboptimal evaluation of direct immunofluorescence.

**Possible Causes of False-Negative Direct Immunofluorescence**
Inadequate, expired transport medium or formalin exposure; Dried sample due to leakage of transport medium or delay in shipping the sample; Inadequate slide transport and storage with exposure to light (photobleaching); Vesiculobullous disorder: - Post-treatment biopsy or patient on immune-modulating treatment; - Biopsy of never-involved skin at distant site; - Completely bullous skin; - Completely detached epidermis; - Erythematous perilesional skin or patient on gluten-free diet (dermatitis herpetiformis); - Ocular mucous membrane pemphigoid. Cutaneous vasculitis: Normal-appearing uninvolved skin. Connective tissue disease: - Early lesion in lupus erythematosus (less than 3 months duration); - ‘Burnt-out’ and treated lesion.
**Possible Causes of False-Positive Direct Immunofluorescence**
Vesiculobullous disorder: - Crush or freezing artifact (pemphigus-like pattern); - Bullous scabies (bullous pemphigoid-like pattern). Cutaneous vasculitis - Distal lower legs near ankles. Connective tissue disorders - Normal sun-exposed skin; - Bullous mastocytosis, granuloma faciale, rosacea; - Formalin exposure.

## Data Availability

Not applicable.
